# Deletion of the c-di-GMP Phosphodiesterase BpdB Attenuates *Brucella melitensis* Virulence by Enhancing Oxidative Stress Sensitivity, Partially via the Host STING Signaling Pathway

**DOI:** 10.3390/ijms27156585

**Published:** 2026-07-24

**Authors:** Na Li, Qiumei Shi, Simin Chen, Yao Feng, Mengsi Li, Yang Li, Shaohui Wang, Yanqing Bao, Jingjing Qi, Tonglei Wu, Mingxing Tian

**Affiliations:** 1 Shanghai Veterinary Research Institute, Chinese Academy of Agricultural Sciences, Shanghai 200241, China; ln2001520@163.com (N.L.); csmin111@163.com (S.C.); 13019329693@163.com (Y.F.); 15236611120@163.com (M.L.); 15255936477@163.com (Y.L.); shwang@shvri.ac.cn (S.W.); ybao@shvri.ac.cn (Y.B.); qijingjing@shvri.ac.cn (J.Q.); 2 Hebei Key Laboratory of Preventive Veterinary Medicine, College of Animal Science and Technology, Hebei Normal University of Science and Technology, Qinhuangdao 066600, China; shiqiumei@126.com

**Keywords:** *Brucella melitensis*, c-di-GMP, BpdB, phosphodiesterase, oxidative stress, STING signaling, intracellular survival, virulence

## Abstract

Brucellosis caused by *Brucella melitensis* is a worldwide zoonotic disease, yet the role of the c-di-GMP phosphodiesterase BpdB in virulence remains incompletely understood. This study aimed to investigate the function of BpdB in *B. melitensis* pathogenicity and its involvement in host STING signaling. A *bpdB* deletion strain (Δ*bpdB*) and a complemented strain (C*bpdB*) were constructed in *B. melitensis* M5. Bacterial growth, stress tolerance, intracellular survival in macrophages, cytokine expression, and mouse virulence were evaluated. Deletion of *bpdB* did not affect in vitro growth but significantly attenuated virulence in BALB/c mice, reducing splenomegaly, splenic bacterial load, and hepatic granuloma formation. The Δ*bpdB* strain exhibited enhanced sensitivity to oxidative stress, whereas resistance to acid, polymyxin B, and SDS remained unchanged. Intracellular survival of Δ*bpdB* in RAW264.7 macrophages was reduced at 72 h post infection, a defect that was completely abrogated in STING-knockout RAW264.7 cells. Δ*bpdB* infection induced higher transcriptional levels of *IFN-β* and *IL-1β*, with *IFN-β* induction strictly dependent on STING. In C57BL/6J mice, the virulence attenuation of Δ*bpdB* was partially STING-dependent, as the reduction in splenic bacterial load was smaller in STING-knockout than in wild-type mice. These findings demonstrate that BpdB contributes to *B. melitensis* virulence by enhancing oxidative stress resistance and dampening STING-dependent host responses, providing new insights into c-di-GMP-mediated host–pathogen interactions.

## 1. Introduction

Brucellosis is a worldwide zoonotic infectious disease caused by bacteria of the genus *Brucella*. The disease poses a serious threat to both animal and human health. In animals, infection primarily manifests as abortion, endometritis, arthritis in pregnant females, and orchitis in males. In humans, brucellosis causes multisystem and multiorgan damage. Early symptoms include undulant fever, fatigue, hyperhidrosis, anorexia, and weight loss. If not treated promptly, the disease may become chronic, presenting as arthritis, osteomyelitis, neurological symptoms, endocarditis, and reproductive disorders, which are difficult to cure [1,2]. Therefore, brucellosis represents a major threat to the healthy development of both humans and animals.

*Brucella* is a Gram-negative coccobacillus that is non-spore-forming, non-motile, and has the characteristics of a facultative intracellular pathogen. Unlike many other pathogenic bacteria, *Brucella* lacks typical virulence factors such as fimbriae, capsules, and exotoxins. Its pathogenicity mainly relies on the ability to invade host cells and survive and replicate intracellularly [3]. To date, the major virulence factors identified in *Brucella* include lipopolysaccharide (LPS), flagella, the type IV secretion system (T4SS), and the two-component regulatory system BvrS/BvrR [3,4]. In recent years, it has been shown that deletion of genes involved in the metabolism of cyclic diguanylate (c-di-GMP) also affects *Brucella* pathogenicity [5,6].

c-di-GMP, discovered three decades ago, is an important bacterial second messenger [7]. It not only regulates a variety of bacterial physiological activities and functions, such as motility, biofilm formation, and virulence factor expression, but also acts as a signaling molecule that directly activates the host STING pathway, inducing type I interferon (IFN-β) expression and NF-κB activation, thereby playing a critical role in host defense against bacterial infection [7,8]. In *Brucella*, the STING signaling pathway also plays an essential role during infection, and this process is independent of the cGAS molecule [9,10]. However, the precise role of bacterial c-di-GMP, as a ligand of STING, in activating the STING pathway during *Brucella* infection remains incompletely understood. c-di-GMP is synthesized by diguanylate cyclases (DGCs) containing GGDEF domains and degraded by specific phosphodiesterases (PDEs) carrying highly conserved EAL or HD-GYP domains [11,12].

In the *Brucella* genome, a total of eleven genes are involved in c-di-GMP metabolism: among them, two genes encode PDE domains, six genes encode DGC domains, and the remaining three genes contain both domains [6]. Three of these genes have been reported to be associated with *Brucella* virulence: *cgsB*, *bpdA*, and *bpdB* [6]. In a *Vibrio* reporter system, CgsB exhibits c-di-GMP synthase activity, whereas BpdA and BpdB display phosphodiesterase activity that degrades c-di-GMP [6]. Infection experiments in IRF-1^−^/^−^ mice revealed that deletion of *cgsB* significantly enhances *Brucella* virulence, whereas deletion of *bpdA* or *bpdB* markedly attenuates the virulence of *B. melitensis* 16M [6]. Further studies have shown that *cgsB* deletion reduces c-di-GMP synthesis in *Brucella*, thereby avoiding excessive activation of the host STING pathway [5,9]. On the other hand, although *bpdA* deletion increases c-di-GMP synthesis, its attenuated virulence is not due to enhanced antimicrobial activity mediated by STING pathway activation [5]. Instead, *bpdA* deletion markedly suppresses the expression of flagellum biosynthesis genes, leads to obvious morphological changes, and renders the bacteria highly sensitive to nutrient-limited conditions, which may be the primary reasons for its attenuated virulence [5]. However, the specific role of BpdB, a c-di-GMP-specific phosphodiesterase, in *Brucella* pathogenesis, and whether it affects virulence through activation of the STING signaling pathway, remains largely unknown.

To investigate the role of BpdB in *Brucella* pathogenesis, we used *B. melitensis* strain M5 as the parental strain and successfully constructed *bpdB* deletion and complemented strains. Through in vitro growth curve analysis, stress tolerance assays, and cell and mouse infection experiments, we systematically evaluated the impact of *bpdB* deletion on *Brucella* virulence and its involvement in activating the host STING signaling pathway during anti-*Brucella* infection. This study clarifies the contribution of BpdB, a c-di-GMP-specific phosphodiesterase, to *Brucella* pathogenicity and demonstrates that the attenuation of virulence caused by *bpdB* deletion is mediated, at least in part, through enhanced susceptibility to oxidative stress and activation of the STING pathway. These findings not only deepen our understanding of the molecular mechanisms underlying *Brucella* virulence but also provide important clues regarding the c-di-GMP-mediated interaction between *Brucella* and the host STING signaling pathway.

## 2. Results

### 2.1. bpdB Deletion and Complemented Strains of B. melitensis Were Successfully Constructed with No Effect on Bacterial Growth

The BpdB protein of *Brucella* contains an EAL domain and exhibits c-di-GMP-specific phosphodiesterase activity [6]. To further explore the function of BpdB, we deleted the complete open reading frame (ORF) of the *bpdB* gene in *B. melitensis* strain M5 using homologous recombination, generating the deletion strain designated Δ*bpdB*. Subsequently, the *bpdB* gene was ectopically complemented in the Δ*bpdB* strain using a miniTn7 transposon, yielding the complemented strain named C*bpdB*.

**Figure 1:**
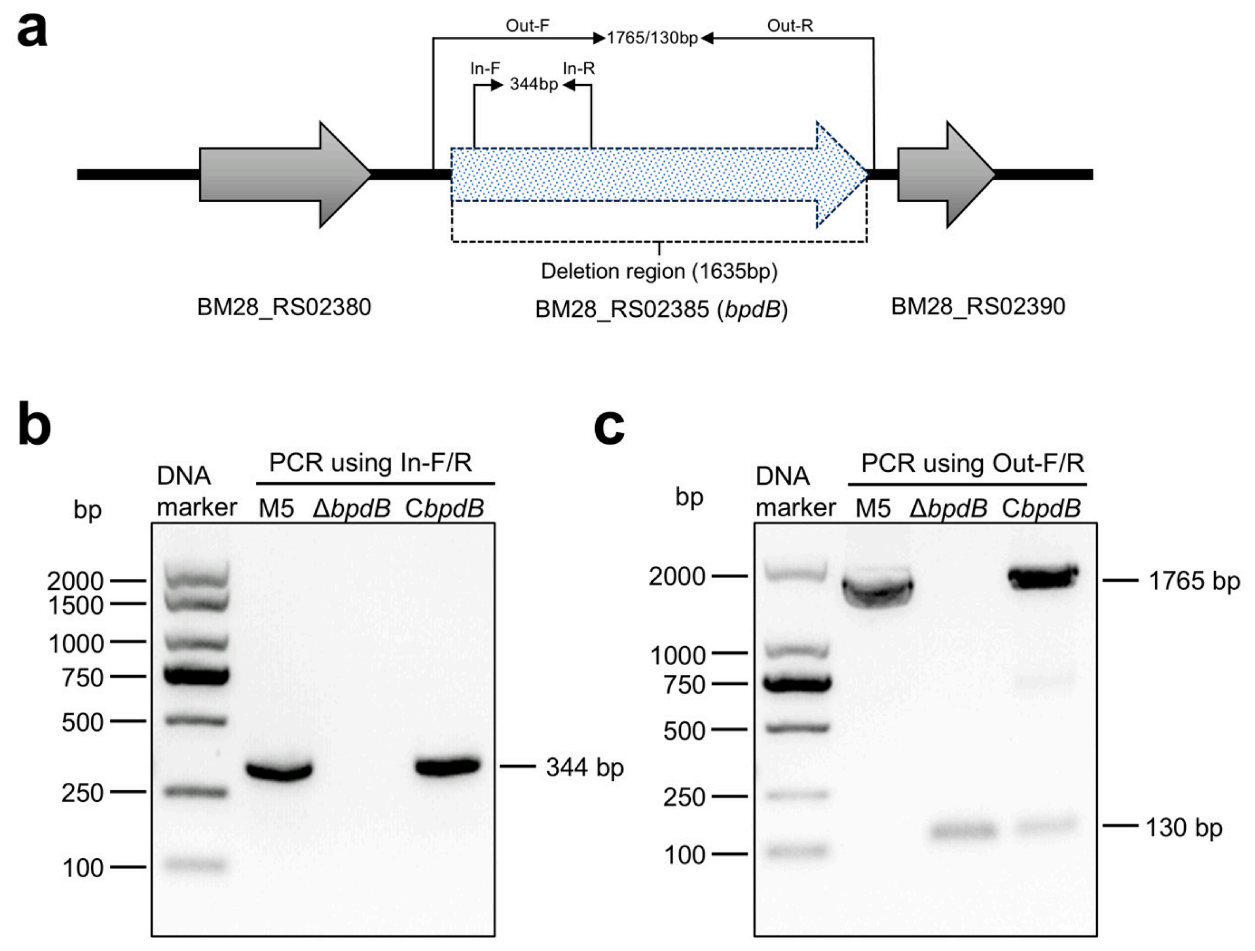
Construction and verification of *bpdB* deletion and complemented strains. (**a**) A Schematic representation of the *bpdB* genomic locus and the binding sites of the primers used. (**b**) PCR verification of the deletion mutant (Δ*bpdB*) and complemented strain (C*bpdB*) using the internal primer pair In-F/R. (**c**) PCR verification of the Δ*bpdB* and C*bpdB* strains using the external primer pair Out-F/R.

The deletion and complemented strains were verified by PCR. Using internal primers In-F/R (primer positions shown in Figure 1a), the M5 parental strain and the C*bpdB* strain produced a specific amplicon of approximately 344 bp, whereas the Δ*bpdB* strain yielded no product (Figure 1b). Using external primers Out-F/R (primer positions shown in Figure 1a), the M5 strain generated a 1765 bp fragment representing the intact *bpdB* gene; the Δ*bpdB* strain produced only a small fragment (~141 bp) due to the deletion; and the C*bpdB* strain simultaneously generated the small deletion-associated fragment (~130 bp) and the full-length *bpdB* fragment (1765 bp) derived from the miniTn7-mediated ectopic complementation (Figure 1c). These results confirm the successful construction of *bpdB* deletion and complementation strains of *B. melitensis*.

**Figure 2:**
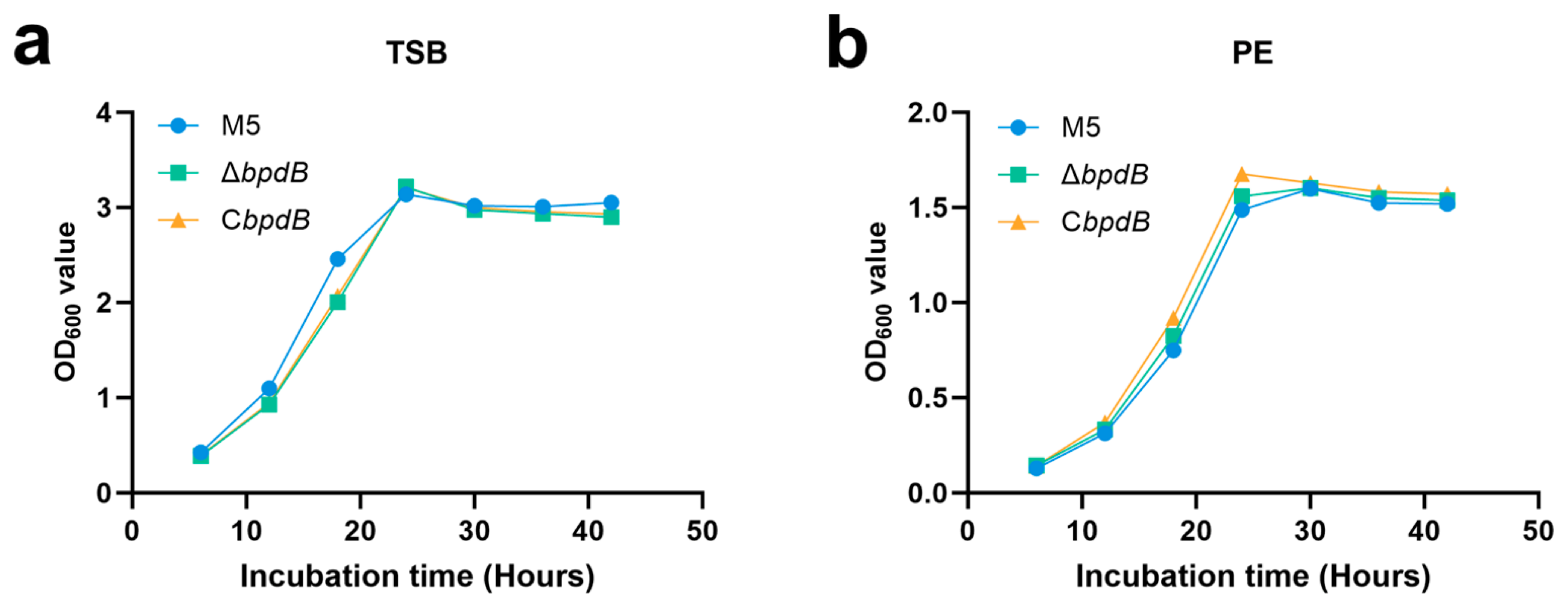
Growth curves of *Brucella* M5 and its derivative strains. (**a**) Growth in tryptic soy broth (TSB). (**b**) Growth in chemically defined PE medium.

To evaluate the effect of *bpdB* deletion on the in vitro growth of *Brucella*, growth curves of the M5 parental strain, Δ*bpdB*, and C*bpdB* were determined in nutrient-rich TSB medium and in chemically defined PE medium. All three strains exhibited similar growth trends in both media (Figure 2a,b). This indicates that *bpdB* deletion does not affect the in vitro growth ability of *Brucella*.

### 2.2. Deletion of bpdB Attenuates the Virulence of B. melitensis in Mice

**Figure 3:**
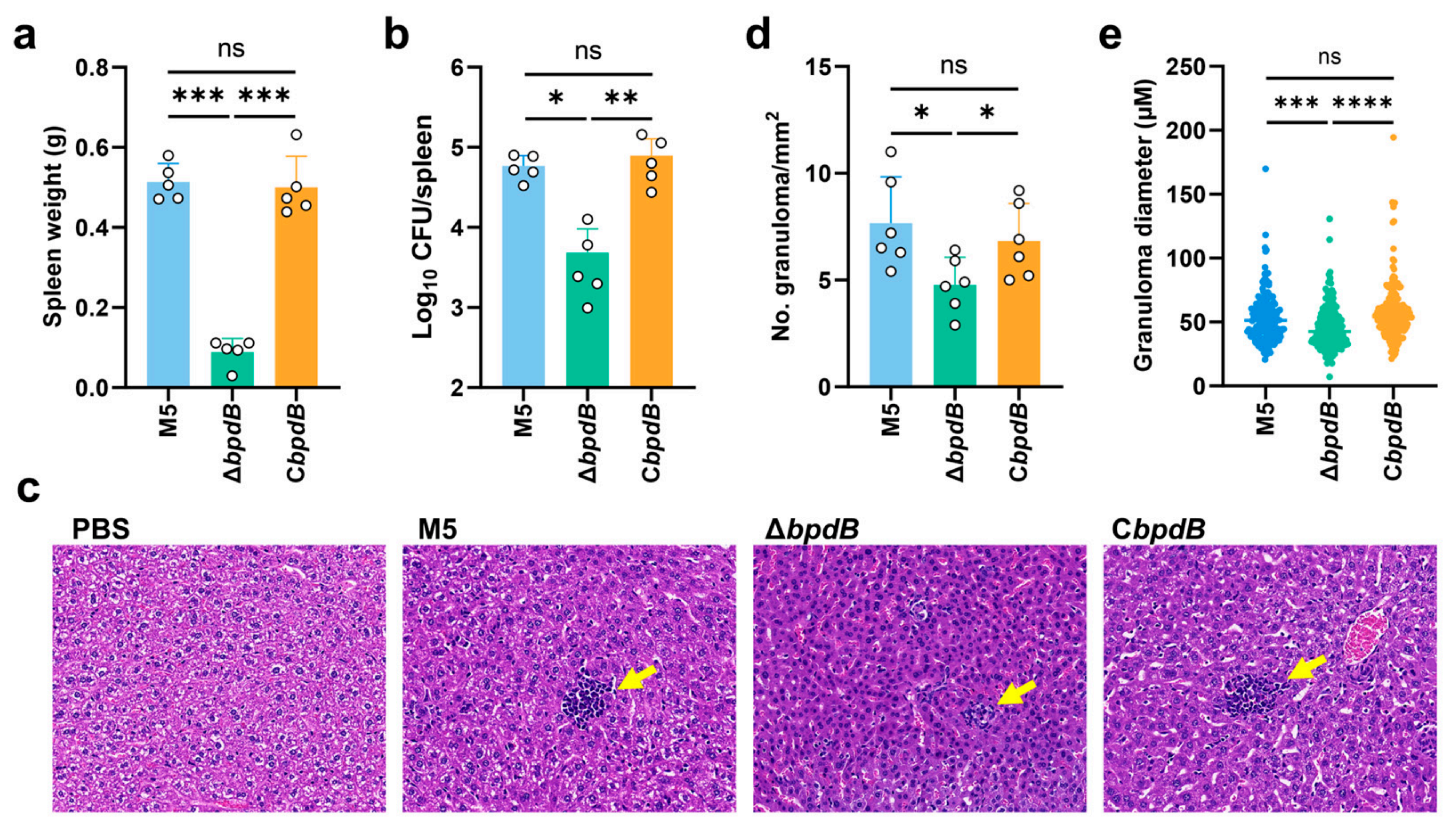
The virulence of *Brucella* strains M5, Δ*bpdB* and C*bpdB* in BALB/c mice. (**a**) Spleen weight at 2 weeks post infection (w.p.i.). (**b**) Bacterial loads in the spleen at 2 w.p.i. (**c**) Histopathological analysis of liver sections. Representative H&E-stained liver tissues at 2 w.p.i. (400× magnification). Yellow arrows indicate granulomas formed by *Brucella* infection in the liver. (**d**) The number of granulomas per mm^2^. (**e**) The diameter of granulomas. Statistical significance was determined using one-way ANOVA followed by Tukey’s multiple comparison test. *, *p* < 0.05; **, *p* < 0.01; ***, *p* < 0.001; ****, *p* < 0.0001; ns, not significant.

To investigate the impact of *bpdB* deletion on *Brucella* virulence, we compared the pathogenicity of the parental strain M5, the deletion strain Δ*bpdB*, and the complemented strain C*bpdB* in BALB/c mice. Two weeks post infection, spleen weight measurements showed that, compared with the M5 group, mice infected with Δ*bpdB* had significantly reduced spleen weights, whereas complementation of *bpdB* (C*bpdB* group) significantly increased spleen weights. This indicates that *bpdB* deletion attenuates the ability of *Brucella* to induce splenomegaly in mice (Figure 3a). Splenic bacterial load analysis revealed that the colonization of Δ*bpdB* in the spleen was significantly lower than that of the parental strain, and the complemented strain restored the bacterial load to a level comparable to that of the parental strain (Figure 3b). Thus, *bpdB* deletion markedly reduces the splenic colonization ability of *Brucella*.

To further assess the effect of *bpdB* deletion on *Brucella* virulence, histopathological changes in the livers of infected mice were analyzed by H&E staining. At two weeks post infection, compared with the PBS control group, all three bacterial strains induced obvious granuloma formation in liver tissues. However, the size of visible granulomas in the Δ*bpdB*-infected livers was significantly smaller than that in the livers infected with the parental and complemented strains (Figure 3c). For quantitative evaluation of granuloma formation, whole-liver panoramic scan images were analyzed using CaseViewer v2.4 software (3DHISTECH Ltd., Budapest, Hungary). The number of granulomas per mm^2^ in liver tissues from Δ*bpdB*-infected mice was significantly lower than that from mice infected with the parental or complemented strain (Figure 3d). Moreover, the mean diameter of granulomas induced by Δ*bpdB* was significantly smaller than that induced by the parental and complemented strains (Figure 3e).

Collectively, these results demonstrate that *bpdB* deletion significantly impairs the ability of *Brucella* to induce splenomegaly, splenic colonization, and hepatic granuloma formation, indicating that loss of *bpdB* substantially reduces the virulence of *Brucella* in mice.

### 2.3. Deletion of bpdB Weakens the Resistance of Brucella to Oxidative Stress

**Figure 4:**
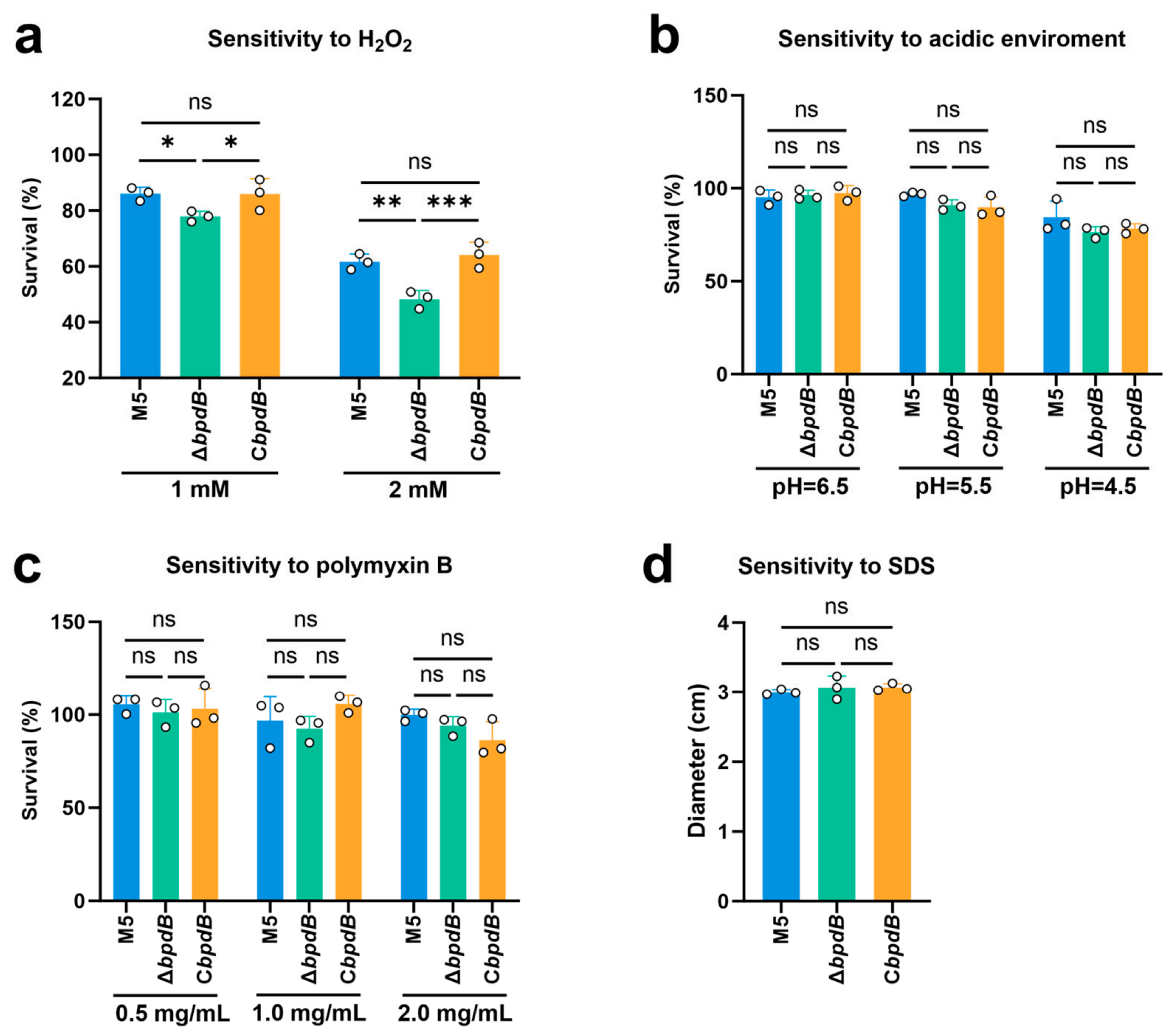
Sensitivity of *Brucella* strains to stress factors. (**a**) Sensitivity to H_2_O_2_. (**b**) Sensitivity to acid shock. (**c**) Sensitivity to polymyxin B. (**d**) Sensitivity to SDS. Statistical significance was determined using two-way ANOVA for (**a**–**c**) and one-way ANOVA for (**d**), followed by Tukey’s multiple comparison test. *, *p* < 0.05; **, *p* < 0.01; ***, *p* < 0.001; ns, not significant.

To explore the mechanism by which *bpdB* deletion attenuates *Brucella* virulence, we systematically evaluated the impact of *bpdB* deletion on the ability of *Brucella* to withstand various environmental stresses. First, the anti-oxidative stress capacity was evaluated by hydrogen peroxide treatment. As shown in Figure 4a, at a final concentration of 1 mM H_2_O_2_, the survival rate of the Δ*bpdB* strain was approximately 78%, while that of the M5 parental strain and the C*bpdB* complemented strain was approximately 86%. Statistical analysis indicated that the survival rate of Δ*bpdB* was significantly lower than that of the parental and complemented strains. At 2 mM H_2_O_2_, the survival rate of Δ*bpdB* was about 48%, whereas those of the parental and complemented strains were about 62%. Again, the survival rate of Δ*bpdB* was significantly lower than that of the other two strains. Furthermore, we assessed the susceptibility of the bacteria to acid stress, the cationic antimicrobial peptide polymyxin B, and the anionic detergent SDS. Under these conditions, the survival ability of the Δ*bpdB* strain showed no significant difference compared with the parental and complemented strains (Figure 4b–d). These results indicate that *bpdB* deletion significantly reduces the resistance of *Brucella* to oxidative stress, but does not affect its sensitivity to acid stress, polymyxin B, or SDS.

### 2.4. Impaired Intracellular Survival of Brucella ΔbpdB Strain Depends on STING Pathway

To investigate the effect of *bpdB* deletion on the ability of *Brucella* to infect host cells, we compared the adhesion, invasion, and intracellular proliferation of the parental strain M5, the deletion strain Δ*bpdB*, and the complemented strain C*bpdB* in HeLa and RAW264.7 cells. In both cells, the adhesion and invasion abilities of the Δ*bpdB* strain were not significantly different from those of the parental and complemented strains (Figure S1).

**Figure 5:**
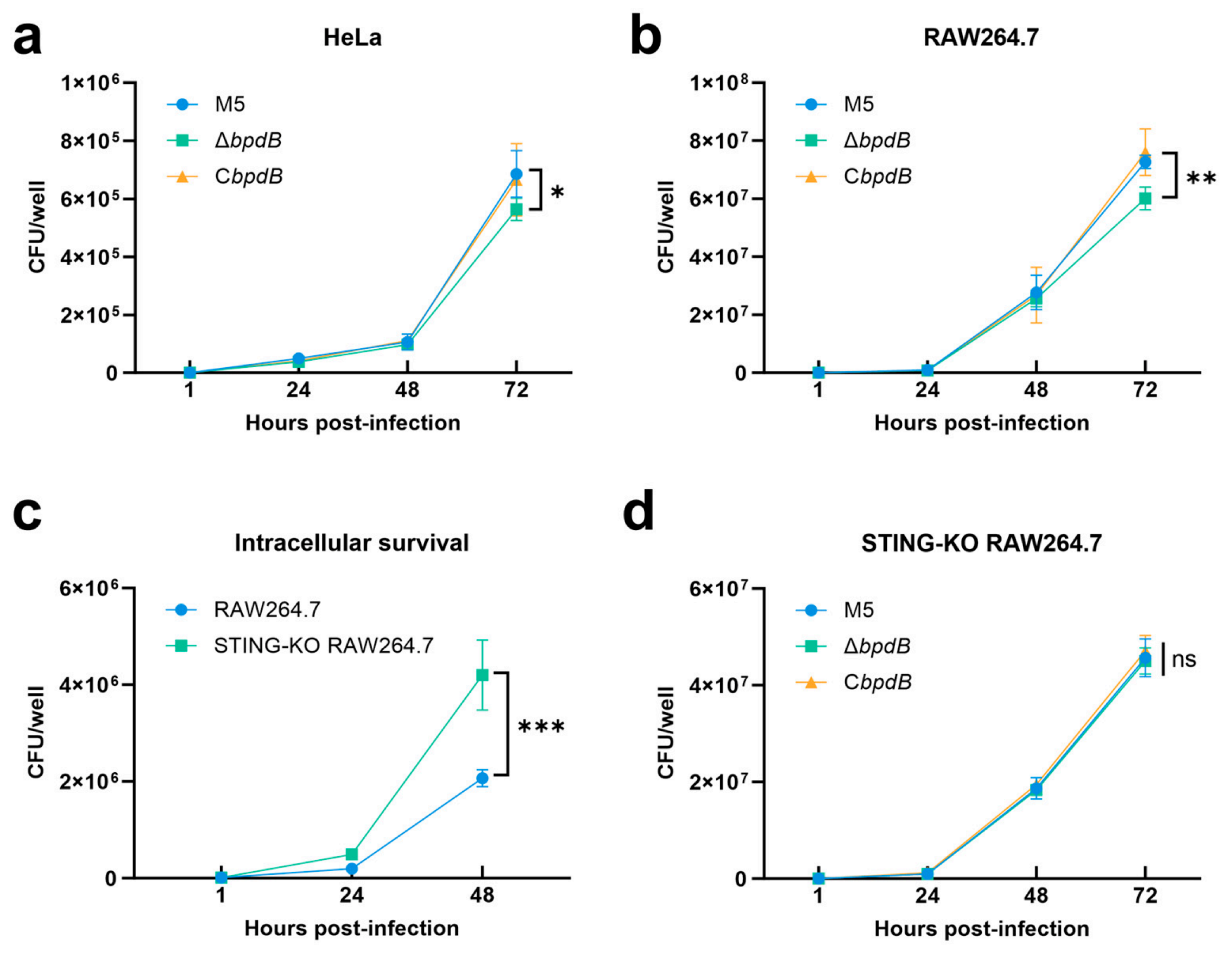
Intracellular survival of *Brucella* strains in host cells. (**a**) Survival of M5, Δ*bpdB* and C*bpdB* strains in HeLa cells. (**b**) Survival of M5, Δ*bpdB* and C*bpdB* strains in RAW264.7 cells. (**c**) Survival of M5 strain in RAW264.7 and STING-KO RAW264.7 cells. (**d**) Survival M5, Δ*bpdB* and C*bpdB* strains in STING-KO RAW264.7 cells. Statistical significance was determined using two-way ANOVA followed by Tukey’s multiple comparison test. *, *p* < 0.05; **, *p* < 0.01; ***, *p* < 0.001; ns, not significant.

The results of intracellular survival showed that in HeLa and RAW264.7 cells, within 48 h of infection, the intracellular bacterial counts of Δ*bpdB* were not significantly different from those of the parental strain. However, at the late time point of 72 h post infection, the intracellular bacterial numbers of Δ*bpdB* were significantly decreased. Complementation of *bpdB* (C*bpdB*) restored the intracellular survival ability at 72 h to the level of the parental strain (Figure 5a,b).

Given that BpdB possesses phosphodiesterase activity and degrades c-di-GMP, a bacterial second messenger that directly activates the host STING signaling pathway, we first assessed the intracellular survival of the *B. melitensis* parental strain M5 in RAW264.7 and STING-knockout (STING-KO) RAW264.7 cells to verify the role of the STING pathway in anti-*Brucella* intracellular survival. As shown in Figure 5c, the intracellular survival of M5 in STING-KO RAW264.7 cells was significantly higher than that in wild-type RAW264.7 cells, indicating that STING plays an important role in restricting intracellular survival of *Brucella*. We next examined whether the reduced intracellular survival of the Δ*bpdB* mutant at later time points was dependent on the STING pathway. To this end, we compared the intracellular survival of the parental, Δ*bpdB*, and complemented strains in STING-KO RAW264.7 cells. Notably, under STING-deficient conditions, no significant differences in intracellular bacterial counts were observed among the three strains at 1, 24, 48, or 72 h post-infection (Figure 5d).

These results indicate that *bpdB* deletion impairs the intracellular survival of *Brucella*, and this impairment depends on the host STING signaling pathway.

### 2.5. ΔbpdB Infection Induces Macrophage IFN-β Expression in a STING-Dependent Manner

To verify whether *bpdB* deletion affects the activation of the host STING signaling pathway by *Brucella* infection, we used qPCR to measure the transcriptional levels of *IFN-β* and *IL-1β* in RAW264.7 and STING-KO RAW264.7 cells infected with the M5 parental strain, Δ*bpdB*, or C*bpdB* for 48 h.

**Figure 6:**
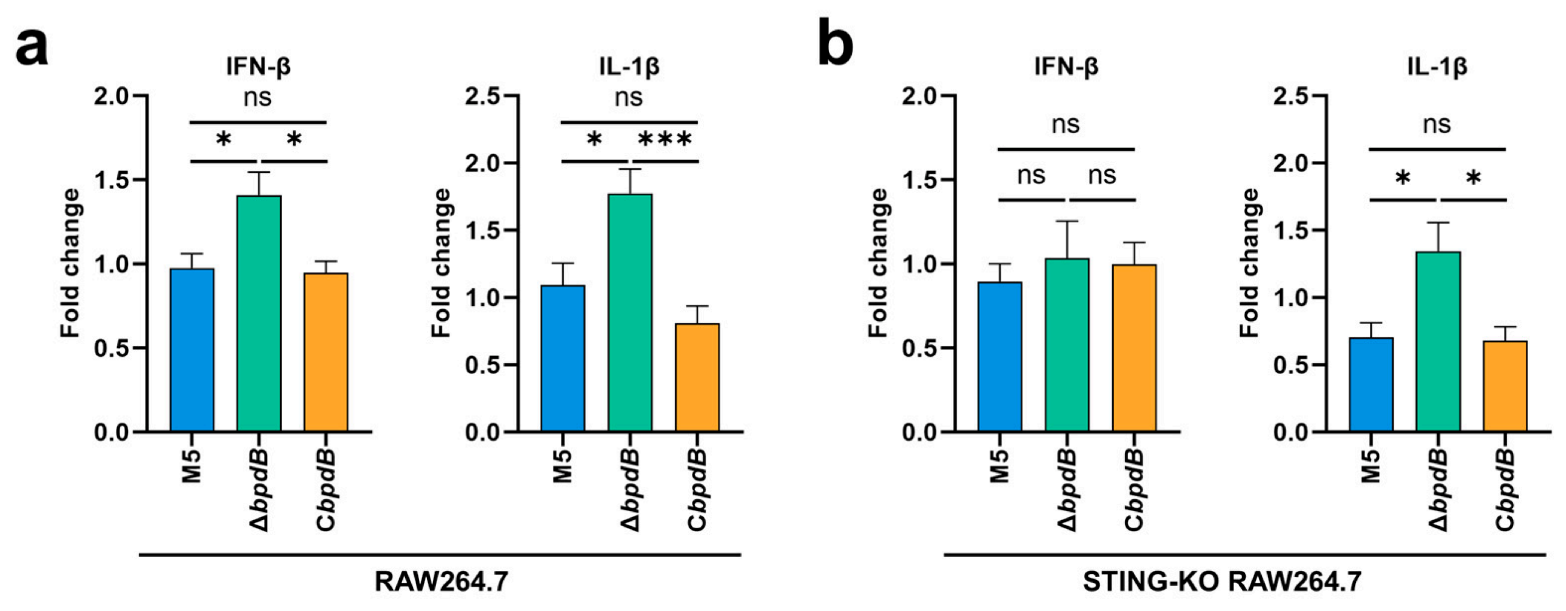
qPCR analysis of IFN-β and IL-1β expression in RAW264.7 and STING-KO RAW264.7 cells infected with M5, Δ*bpdB*, or C*bpdB* at 48 h post infection. (**a**) RAW264.7 cells. (**b**) STING-KO RAW264.7 cells. Statistical significance was determined using one-way ANOVA followed by Tukey’s multiple comparison test. *, *p* < 0.05; ***, *p* < 0.001; ns, not significant.

In RAW264.7 cells, infection with Δ*bpdB* significantly increased the transcript levels of *IFN-β* and *IL-1β* compared with infection with the parental strain; complementation of *bpdB* (C*bpdB*) restored the expression of both cytokines to the levels observed in the parental strain (Figure 6a).

To further evaluate whether the induction of *IFN-β* and *IL-1β* by Δ*bpdB* infection depends on the STING pathway, we performed qPCR analysis of these cytokines in STING-KO RAW264.7 cells following *Brucella* infection. In STING-deficient cells, the upregulation of *IFN-β* expression by Δ*bpdB* was abolished, and no significant difference was found among the three strains. In contrast, the Δ*bpdB*-induced *IL-1β* expression remained significantly higher than that induced by the parental and complemented strains (Figure 6b).

These results demonstrate that Δ*bpdB* infection significantly activates the expression of *IFN-β* and *IL-1β* in macrophages; among them, *IFN-β* expression is strictly dependent on the STING signaling pathway, whereas *IL-1β* expression is only partially dependent on this pathway.

### 2.6. The Attenuated Virulence of the ΔbpdB Strain in Mice Depends Partially on the STING Signaling Pathway

**Figure 7:**
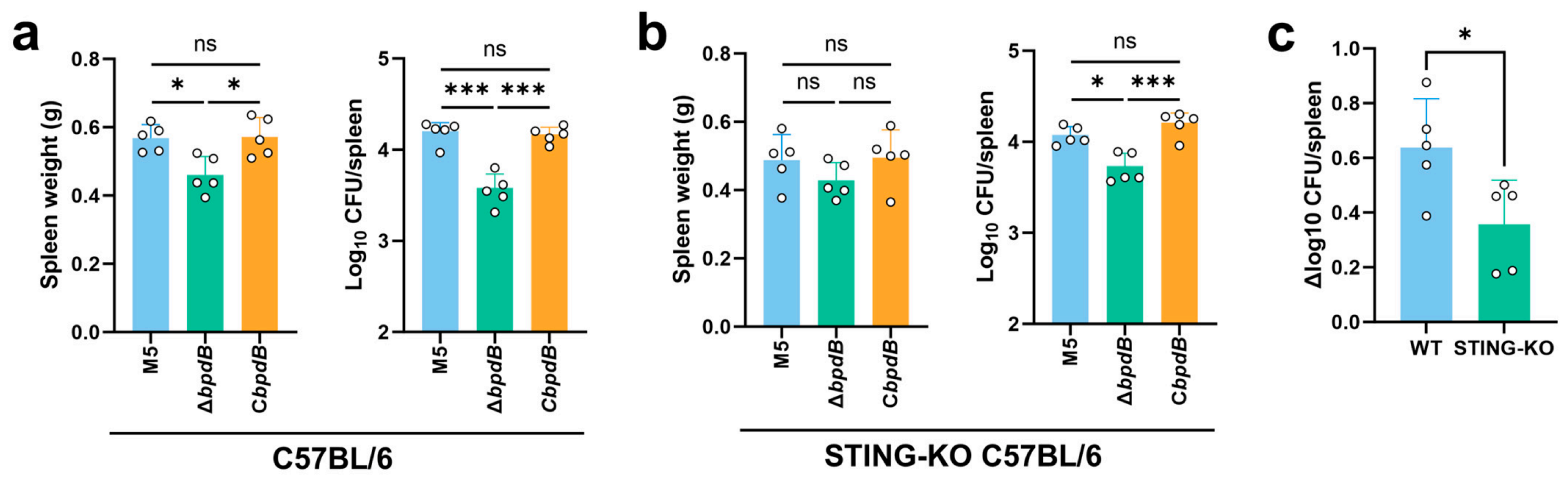
Comparison of virulence of *Brucella* strains M5, Δ*bpdB* and C*bpdB* in WT and STING-KO mice. (**a**) Spleen weight and splenic bacterial loads in infected C57BL/6J WT mice. (**b**) Spleen weight and splenic bacterial loads in infected STING-KO C57BL/6J mice. (**c**) Reduction in splenic bacterial load of Δ*bpdB* relative to M5 in WT and STING-KO mice. Statistical significance was determined using one-way ANOVA followed by Tukey’s multiple comparison test. *, *p* < 0.05; ***, *p* < 0.001; ns, not significant.

To explore whether the attenuated virulence of the Δ*bpdB* strain depends on activation of the host STING signaling pathway, we compared the infection phenotypes of the parental strain M5, Δ*bpdB*, and C*bpdB* in C57BL/6J wild-type (WT) and STING-KO C57BL/6J mice. At 10 days post infection, spleen weight measurements showed that in WT mice, Δ*bpdB* infection led to a significantly reduced spleen weight compared with M5 or C*bpdB* infection. The splenic bacterial load of Δ*bpdB* was also significantly lower than that of the parental and complemented strains (Figure 7a). In STING-KO mice, although the spleen weight of the Δ*bpdB*-infected group was lower than that of the M5 and C*bpdB* groups, the difference was not statistically significant. However, the splenic bacterial load of Δ*bpdB* remained significantly lower than that of M5 and C*bpdB* in STING-KO mice (Figure 7b). Notably, the mean reduction in splenic bacterial load of Δ*bpdB* compared with the parental strain was 0.67 log in WT mice but only 0.36 log in STING-KO mice, and the extent of reduction was significantly smaller in STING-KO mice than in WT mice (Figure 7c). Taken together, these results indicate that the attenuation of pathogenicity in mice caused by Δ*bpdB* is only partially mediated by the STING signaling pathway.

## 3. Discussion

In this study, we systematically investigated the role of BpdB, a c-di-GMP-specific phosphodiesterase, in the pathogenicity of *B. melitensis* and its involvement in modulating the host STING signaling pathway. We found that *bpdB* gene deletion did not affect in vitro bacterial growth but significantly attenuated bacterial virulence in mice; the Δ*bpdB* strain exhibited enhanced sensitivity to oxidative stress, while its resistance to acid stress, polymyxin B, and SDS remained unchanged. At the late stage of cell infection (72 h post infection), the intracellular survival ability of the Δ*bpdB* strain was markedly reduced, and this defect was completely abrogated in STING-KO macrophages. Moreover, Δ*bpdB* infection induced elevated transcriptional levels of *IFN-β* and *IL-1β* in macrophages, with *IFN-β* induction being strictly dependent on the STING pathway. In a mouse model, the virulence attenuation of the Δ*bpdB* strain was partially dependent on the STING signaling pathway. Together, these results demonstrate that BpdB contributes to *Brucella* pathogenicity by modulating oxidative stress resistance and STING-dependent host responses, providing new insights into the c-di-GMP-mediated interplay between *Brucella* and the host.

A previous study reported that deletion of *bpdA* in *B. melitensis* 16M affects bacterial adaptation to low-nutrient conditions [5]. Given this finding, we asked whether BpdB, another c-di-GMP phosphodiesterase, might also play a role in bacterial growth and nutrient adaptation. To address this question, we evaluated the growth of the Δ*bpdB* strain in both nutrient-rich TSB medium and chemically defined low-nutrient PE medium. Our results showed that deletion of *bpdB* in the M5 strain did not impair bacterial growth under either condition. This indicates that, in contrast to BpdA, BpdB is not essential for basic metabolism or replication in vitro. Virulence attenuation without compromised growth fitness is common among *Brucella* virulence factors, such as T4SS or LPS mutants [13,14], suggesting that BpdB primarily participates in host adaptation and intracellular survival rather than affecting fundamental growth metabolism. Nevertheless, despite the lack of growth impairment, the Δ*bpdB* strain exhibited significant differences in multiple virulence-related phenotypes.

One of the most prominent phenotypic changes in the Δ*bpdB* strain was its increased susceptibility to hydrogen peroxide. Oxidative stress is a key host defense mechanism employed by macrophages to eliminate intracellular pathogens, and *Brucella* has evolved multiple strategies to resist oxidative killing, including the production of catalase (KatE), superoxide dismutase (SodC), and alkyl hydroperoxide reductase (AhpC) [15,16]. The enhanced H_2_O_2_ sensitivity of the Δ*bpdB* strain suggests that BpdB, through its phosphodiesterase activity regulating c-di-GMP levels, may directly or indirectly influence the expression or activity of these anti-oxidant systems. Notably, the Δ*bpdB* strain showed no altered sensitivity to acid stress, polymyxin B, or SDS, indicating that the effect of *bpdB* deletion is relatively specific to oxidative stress rather than globally compromising cell envelope integrity or general stress responses. This specificity further supports the notion that BpdB-mediated c-di-GMP signaling is linked to a specific stress response pathway in *Brucella*. Whether this enhanced oxidative stress sensitivity alone is sufficient to explain the intracellular and in vivo virulence attenuation of the Δ*bpdB* strain is addressed by our cellular experiments, which provided further mechanistic clues.

Intracellular survival and proliferation assays revealed that the Δ*bpdB* strain displayed normal bacterial loads during early infection (within 48 h) but showed a significant reduction at the late stage (72 h). This late-phase defect suggests that BpdB is particularly important for sustained intracellular replication or for counteracting host defenses that become activated at later stages of infection. The observation that this defect was completely abrogated in STING-KO RAW264.7 cells provides strong evidence that the reduced intracellular fitness of the Δ*bpdB* strain is mediated by the host STING signaling pathway. Because BpdB degrades c-di-GMP, *bpdB* deletion may lead to elevated intracellular c-di-GMP levels in *Brucella*. Elevated c-di-GMP is likely recognized by the host STING pathway, as STING is a direct innate immune sensor of bacterial cyclic dinucleotides [7,8]. Indeed, we found that Δ*bpdB* infection induced significantly higher *IFN-β* transcription in a STING-dependent manner, consistent with the hypothesis that increased c-di-GMP production in the Δ*bpdB* strain leads to STING hyperactivation, thereby enhancing type I interferon responses [8]. Type I interferons exhibit a dual role in bacterial infections: in some contexts, they restrict bacterial replication, while in others they may promote infection [17,18]. In *Brucella* infection, it has been reported that STING-mediated IFN-β production contributes to host resistance [9]. Thus, the reduced intracellular survival of the Δ*bpdB* strain is likely due to enhanced host antimicrobial responses triggered by elevated c-di-GMP-STING signaling.

At the cytokine regulation level, we observed a noteworthy phenomenon: although *bpdB* deletion upregulated both *IFN-β* and *IL-1β* expression in macrophages, the regulation of these two cytokines differed in their dependence on STING. *IFN-β* induction by Δ*bpdB* was completely abolished in STING-KO cells, indicating strict STING dependency. In contrast, *IL-1β* upregulation was only partially reduced in the absence of STING, suggesting that additional STING-independent pathways also contribute to *IL-1β* expression. This is consistent with the known regulation of *IL-1β*, which typically involves NF-κB activation downstream of multiple pattern recognition receptors (such as TLRs) and the inflammasome [19,20]. The partial STING dependence of *IL-1β* may reflect the convergence of STING-dependent and STING-independent signals. Whether these in vitro differential cytokine expression patterns truly reflect the in vivo virulence attenuation mechanism was addressed by our mouse experiments.

Using WT and STING-KO mice, our in vivo experiments revealed a more nuanced picture. In both WT and STING-KO mice, the splenic bacterial load of the Δ*bpdB* strain was significantly lower than that of the parental strain, but the magnitude of reduction was significantly smaller in STING-KO mice (0.36 log) than in WT mice (0.67 log). This indicates that the STING pathway substantially, but not completely, participates in the virulence attenuation of the Δ*bpdB* strain. The residual virulence attenuation observed in STING-KO mice (approximately 0.36 log) suggests that STING-independent mechanisms also play a role. One likely candidate mechanism is the enhanced oxidative stress sensitivity of the Δ*bpdB* strain. Even in the absence of STING signaling, host phagocytes still produce reactive oxygen species via NADPH oxidase [21], and the inherent susceptibility of the Δ*bpdB* strain to oxidative killing could directly limit its in vivo survival. Therefore, the virulence attenuation of the Δ*bpdB* strain appears to be multifactorial, involving both STING-dependent immune activation and STING-independent intrinsic stress sensitivity.

Comparing BpdB with other c-di-GMP-metabolizing enzymes in *Brucella* helps to further understand its function. Deletion of *cgsB* (a diguanylate cyclase) reduces c-di-GMP synthesis and enhances virulence, possibly by avoiding excessive STING activation [6,9]. In contrast, deletion of another PDE, *bpdA*, also attenuates virulence, but primarily through effects on flagellar gene expression and nutrient stress sensitivity rather than STING activation [5]. The present study positions BpdB as a PDE whose deletion leads to virulence attenuation through two distinct mechanisms: enhanced oxidative stress sensitivity and STING-dependent host activation. These findings highlight the functional diversity among c-di-GMP metabolic enzymes in *Brucella* despite their shared substrate. The net effect of perturbing c-di-GMP homeostasis likely depends on the specific spatial, temporal, and quantitative changes in c-di-GMP levels, as well as on the downstream effector pathways each enzyme is coupled to.

Several limitations of this study should be acknowledged. First, we did not directly measure intracellular c-di-GMP levels in the Δ*bpdB* strain, nor did we quantify c-di-GMP accumulation during infection. Future studies using biosensors or LC-MS/MS would help establish the direct link between BpdB activity, c-di-GMP pools, and the observed phenotypes. Second, the molecular mechanism by which BpdB regulates oxidative stress resistance remains unknown; whether BpdB directly controls the expression of anti-oxidant genes or influences other stress response regulators requires further investigation. Third, while we demonstrated that STING is involved in the reduced intracellular survival of Δ*bpdB*, the downstream effector molecules responsible for bacterial clearance (e.g., IFN-β, IL-1β, or other STING-regulated genes) have not been functionally dissected. Knockout of *IFN-β* or its receptor in macrophages or mice could clarify the causal role of type I interferons. Finally, the partial STING dependence observed in vivo suggests the existence of other host pathways that cooperate with STING; identifying these pathways will enable a more comprehensive understanding of the host–pathogen interaction.

In summary, this study demonstrates that the c-di-GMP phosphodiesterase BpdB is an important virulence determinant in *B. melitensis*. *bpdB* deletion does not affect bacterial in vitro growth but attenuates virulence in mice through two complementary mechanisms: (1) enhanced sensitivity to oxidative stress, and (2) increased activation of the host STING signaling pathway, thereby enhancing host antimicrobial responses. The STING pathway is required for the late-phase intracellular survival defect of the Δ*bpdB* strain in macrophages and plays an important (albeit incomplete) role in in vivo virulence attenuation. These findings not only deepen our understanding of the molecular mechanisms underlying *Brucella* virulence but also provide important clues regarding the c-di-GMP-mediated interplay between *Brucella* and the host STING signaling pathway. BpdB may represent a potential target for the development of attenuated vaccines or novel therapeutic strategies against brucellosis.

## 4. Materials and Methods

### 4.1. Bacterial Strains and Cell Lines

The attenuated vaccine strain *B. melitensis* M5 used in this study was purchased from the China Veterinary Culture Collection Center (CVCC, Beijing, China). *Brucella* strains and their derivatives were cultured in TSA or TSB medium (Difco, Franklin Lakes, NJ, USA) at 37 °C in a 5% CO_2_ atmosphere. All manipulations involving *B. melitensis* M5 and its derivatives were performed in a biosafety level 2 laboratory at the Shanghai Veterinary Research Institute. *Escherichia coli* DH5α (TIANGEN, Beijing, China) was cultured in LB medium. The mouse monocyte/macrophage cell line RAW264.7 (ATCC TIB-71, Manassas, VA, USA), the STING-KO RAW264.7 cell line (Cyagen Biosciences, Suzhou, China), and the human cervical carcinoma cell line HeLa (ATCC CCL-2, Manassas, VA, USA) were maintained in DMEM (Gibco, Grand Island, NY, USA) supplemented with 10% fetal bovine serum (ExCell Bio, Suzhou, China) at 37 °C in a 5% CO_2_ atmosphere. The bacterial strains and plasmids used in this study are listed in Table 1.

### 4.2. Plasmid Construction

The suicide plasmid was constructed as previously described by our group [22]. Briefly, using the *B. melitensis* M5 genome as a template, the upstream and downstream homology arm fragments of the *bpdB* gene were amplified by PCR with primer pairs BpdB-UF/UR and BpdB-DF/DR, respectively. The upstream and downstream fragments were then fused by overlap PCR using primers BpdB-UF and BpdB-DR. The fused fragment was ligated into the linearized pKB vector using a homologous recombination cloning kit (Vazyme, Nanjing, China) to generate the suicide plasmid pKB-Δ*bpdB*.

The complemented plasmid was constructed following the same method previously described by our group [22], which is based on the miniTn7 transposon system for site-directed insertion of the complementation fragment into a specific chromosomal locus. Specifically, the complementation fragment was amplified by PCR from the M5 genome using primers CbpdB-F/R. The purified fragment was then recombined with the linearized pMiniTn7TK vector to generate the complemented plasmid pMiniTn7TK-C*bpdB*. The primers used above are listed in Table 2.

### 4.3. Construction of Deletion and Complemented Strains

*B. melitensis* M5 was cultured to the logarithmic growth phase (OD_600_ = 0.6–0.8). The bacterial suspension was incubated on ice for 30 min, washed twice with pre-chilled sterile deionized water, and resuspended in pre-chilled 10% glycerol to prepare electrocompetent cells. One hundred microliters of competent cells was mixed with 1 μg of the suicide plasmid pKB-Δ*bpdB* and incubated on ice for 10 min. The mixture was transferred to a pre-chilled electroporation cuvette (1 mm gap, Bio-Rad, Hercules, CA, USA) and electroporated at 2400 V, 25 μF, and 400 Ω. Immediately after electroporation, pre-warmed TSB medium (37 °C) was added, and the cells were incubated with shaking at 37 °C for 8 h. An appropriate volume of the culture was plated onto TSA plates containing kanamycin and incubated at 37 °C until single colonies appeared to complete the first round of homologous recombination screening. Single colonies were picked, inoculated into TSB, and cultured for 24 h, after which they were plated onto TSA plates containing 5% sucrose for the second round of negative selection. Single colonies were subjected to PCR identification, and the successfully constructed deletion strain was designated Δ*bpdB*.

To construct the complemented strain, electrocompetent cells of the Δ*bpdB* strain were prepared as described above. One microgram of the complemented plasmid pMiniTn7TK-C*bpdB* and 1 μg of the helper plasmid pHelp1 were co-electroporated into the Δ*bpdB* strain. After the addition of pre-warmed TSB medium and incubation with shaking at 37 °C for 8 h, an appropriate volume of the culture was plated onto TSA plates containing kanamycin for selection. Single colonies were picked, cultured in TSB, and identified by PCR. The successfully constructed complementation strain was designated C*bpdB*.

### 4.4. Growth Curve Determination

Logarithmic-phase cultures of M5, Δ*bpdB*, and C*bpdB* were inoculated into TSB or PE medium (composition: 2.3 g/L K_2_HPO_4_, 3 g/L KH_2_PO_4_, 0.1 g/L Na_2_S_2_O_3_·5H_2_O, 5 g/L NaCl, 0.2 g/L nicotinic acid, 0.2 g/L thiamine, 0.07 g/L pantothenic acid, 0.5 g/L (NH_4_)_2_SO_4_, 0.01 g/L MgSO_4_, 0.1 mg/L MnSO_4_, 0.1 mg/L FeSO_4_, 0.1 mg/L biotin, 0.15 g/L methionine, 2 g/L erythritol) [23]. The initial OD_600_ was adjusted to 0.1, and the cultures were incubated at 37 °C with shaking at 200 rpm. OD_600_ was measured every 6 h for a total of 42 h. Growth curves were plotted based on the measurements.

### 4.5. Sensitivity to Stress Factors

*B. melitensis* M5, Δ*bpdB*, and C*bpdB* were cultured to the logarithmic growth phase, and the OD_600_ was measured. The bacterial concentration was adjusted to 5 × 10^5^ CFU/mL. Fifty microliters of the bacterial suspension was mixed with an equal volume of polymyxin B (final concentrations of 0.5, 1.0, and 2.0 mg/mL) or H_2_O_2_ (final concentrations of 1 mmol/L and 2 mmol/L), with a PBS-treated group serving as the control. After incubation at 37 °C with shaking for 1 h, the cultures were serially diluted 10-fold, plated onto TSA plates, and viable CFUs were counted. In parallel, 50 μL of the diluted bacterial suspension was inoculated into 0.1% peptone water at pH 7.2, 6.5, 5.5, and 4.5. After thorough mixing, the cultures were incubated at 37 °C with shaking for 2 h, using the pH 7.2 group as the control. After incubation, the bacterial cultures were serially diluted 10-fold, and 100 μL of each dilution was plated onto TSA plates. The survival rate was calculated using the following formula: Survival rate (%) = (CFU of treatment group/CFU of control group) × 100%. Three biological replicates were performed for each group.

Additionally, bacterial sensitivity to SDS was determined using the disk diffusion method. One hundred microliters of M5, Δ*bpdB*, and C*bpdB* cultures at an OD_600_ of approximately 1.0 was evenly spread onto TSA plates. A sterile 7 mm diameter blank disk was placed in the center of each plate using sterile forceps, and 7 μL of 20% SDS solution was added to the disk. Each experiment was performed in triplicate. The plates were incubated at 37 °C in a 5% CO_2_ atmosphere for a standardized period of 96 h (4 days). Following incubation, the diameters of the inhibition zones were accurately measured using a vernier caliper.

### 4.6. Cell Infection Assays

RAW264.7 or STING-KO RAW264.7 cells were seeded at a density of 2.5 × 10^5^ cells/well in 24-well plates, while HeLa cells were seeded at 7 × 10^4^ cells/well in 24-well plates and cultured to confluence. The multiplicity of infection (MOI) values were independently optimized based on the distinct phagocytic capacities of the two cell types: an MOI of 100:1 was used for RAW264.7 and STING-KO RAW264.7 cells, and an MOI of 500:1 for HeLa cells [24]. The M5, Δ*bpdB*, and C*bpdB* strains were used to infect cells at the corresponding MOIs. The plates were centrifuged at 400× *g* for 5 min to promote uniform bacterial contact with the cells, followed by incubation at 37 °C for 1 h to allow for adhesion, at which point the infection time was designated as 0 h. After washing three times with PBS, 200 μL of PBS containing 0.25% Triton X-100 was added to each well to lyse the cells for 15 min. The lysates were plated onto TSA plates and incubated at 37 °C for 3–5 days for CFU enumeration, which represented the number of bacteria adherent to the cells. The remaining infected cells were replaced with DMEM containing 100 μg/mL gentamicin to kill extracellular bacteria. After three washes with PBS, the cells were lysed and CFU were counted as described above, representing the number of internalized bacteria. The remaining cells were maintained in DMEM containing 20 μg/mL gentamicin and 2% FBS. At 1, 24, 48, and 72 h post infection, cells were lysed and CFU were enumerated by the same method to generate intracellular survival curves.

### 4.7. Total RNA Extraction and qPCR Analysis

RAW264.7 cells were infected with the M5, Δ*bpdB*, and C*bpdB* strains. At 24 h and 48 h post infection, total RNA was extracted using the VeZol-Pure Total RNA Isolation Kit (Vazyme, Nanjing, China), and genomic DNA was removed using the Turbo DNA-free Kit (Thermo Fisher, Waltham, MA, USA). Reverse transcription was performed using the PrimeScript RT Kit (Takara, Kusatsu, Japan) to synthesize cDNA. Using the cDNA as a template, the transcriptional levels of *IFN-β* and *IL-1β* were measured by qPCR using the ChamQ Universal SYBR qPCR Master Mix (Vazyme, Nanjing, China) on a QuantStudio Real-Time PCR System (Thermo Fisher, Waltham, MA, USA). *β-Actin* was used as an internal control for data normalization, and the relative transcription levels were calculated using the 2^−ΔΔCt^ method. Three biological replicates were performed for each sample.

### 4.8. Mouse Infection Experiments

Six- to eight-week-old female BALB/c mice were purchased from Shanghai Jiesijie Laboratory Animal Co., Ltd. (Shanghai, China). All animal experiments were approved by the Institutional Animal Care and Use Committee of the Shanghai Veterinary Research Institute and were conducted in an animal biosafety level 2 facility (Approval Number: SV-20251226-02). M5, Δ*bpdB*, and C*bpdB* strains were cultured in TSB to the logarithmic growth phase, adjusted to an OD_600_ of 1.0 (approximately 5 × 10^9^ CFU/mL), and diluted with PBS to 1 × 10^7^ CFU/mL. BALB/c mice were infected via intraperitoneal injection with 100 μL of the bacterial suspension (1 × 10^6^ CFU), with five mice per group. Mice injected with PBS served as the control group. Two weeks post infection, mice were euthanized by cervical dislocation. Spleens were aseptically collected and weighed. After homogenization in 3 mL of PBS containing 0.25% Triton X-100, serial 10-fold dilutions were plated onto TSA plates, and CFU were counted. The bacterial load was calculated as CFU per gram of spleen. Liver tissues from M5-, Δ*bpdB*-, and C*bpdB*-infected mice were collected and fixed in 4% paraformaldehyde for at least 24 h. Paraffin embedding, sectioning, and H&E staining were performed by Wuhan Boerfu Biotechnology Co., Ltd. (Wuhan, China). Whole-slide images were acquired using the 3DHISTECH Pannoramic whole-slide scanner system (3DHISTECH) and analyzed using CaseViewer v2.4 software (3DHISTECH). Six random fields of view (each with an area of 10 mm^2^) per group were selected for quantification of granuloma numbers and measurement of granuloma diameters, followed by statistical analysis.

To evaluate the relationship between the pathogenicity of *B. melitensis* M5, Δ*bpdB*, and C*bpdB* and the host STING signaling pathway, we further performed virulence assessments using C57BL/6J WT and STING-KO C57BL/6J mice. Six- to eight-week-old female C57BL/6J WT and STING-KO mice were bred and maintained at the Shanghai Veterinary Research Institute. Five mice per group were intraperitoneally injected with 1 × 10^6^ CFU of the bacterial suspension. On day 10 post infection, mice were euthanized, and spleens were collected, weighed, and processed for bacterial load determination as described above. The contribution of the STING signaling pathway to the attenuation of Δ*bpdB* virulence was further analyzed by comparing the reduction in splenic bacterial load of Δ*bpdB* relative to the parental M5 strain in WT and STING-KO mice.

### 4.9. Statistical Analysis

Data analysis was performed using GraphPad Prism 9.0 software (GraphPad Software, San Diego, CA, USA). Comparisons among multiple groups were conducted using one-way or two-way analysis of variance (ANOVA) followed by Tukey’s multiple comparisons test. Statistical significance was set at *p* < 0.05.

## Figures and Tables

**Table 1:** The bacterial strains and plasmids used in this study.

Strains and Plasmids	Description	Sources
Strains		
*B. melitensis* M5	Attenuated strain; smooth phenotype	CVCC
Δ*bpdB*	The *bpdB* deletion mutant deprived from M5	This study
C*bpdB*	The *bpdB* complemented strain	This study
*E. coli* DH5α	F−, φ80d*lacZ* ΔM15, Δ*(lacZYA-argF)*U169, *recA1*, *endA1*, *hsdR17(rk−, mk+)*, *phoA*, *supE44*, *thi-1*, *gyrA96*, *relA1*, λ−	TIANGEN
Plasmids		
pKB	Kan^R^; SacB	[22]
pMiniTn7TK	Amp^R^, Kan^R^; miniTn7 transposon	[22]
pHelp1	Amp^R^; Tn7 transposase TnsABCD	[22]
pKB-Δ*bpdB*	pKB plasmid carrying the upstream and downstream homologous fragments of the *bpdB* gene	This study
pMiniTn7TK-C*bpdB*	pMiniTn7TK carrying the *bpdB* gene with its promoter and terminator regions	This study

**Table 2:** The primers used in this study.

Primers	Sequence (5′-3′)	Description
BpdB-UF	GGTACCCGGGGATCCGGGCTTGCTATTGGAAAATG	PCR amplification of *bpdB* upstream homologous fragment
BpdB-UR	TCCGTGCGGCCCGCCTATATGTCCACCTAAAATTC	
BpdB-DF	TAGGTGGACATATAGGCGGGCCGCACGGAAAATAC	PCR amplification of *bpdB* downstream homologous fragment
BpdB-DR	TGCCTGCAGGTCGACTGGTCGATTCCGAGATCATC	
CbpdB-F	CATGAGCTCACTAGTGGATCCAGGATGCGCGACATGTTTCG	PCR amplification of *bpdB* complemented fragment
CbpdB-R	GCAAGGCCTTCGCGAGGTACCAGGCGCAGAATAACCCAAAC	
In-F	CAACTGGTCGGAAAAGGACT	Internal identification primers for the *bpdB* mutant
In-R	GGCGTTATCAAGGCATCATC	
Out-F	GAGTTCGGAATCGCTATGAC	External identification primers for the *bpdB* mutant
Out-R	CTCAAAACTGCTCCACTCGC	
RT-ActB-F	CATTGCTGACAGGATGCAGAAGG	qPCR for the *β-actin* gene
RT-ActB-R	TGCTGGAAGGTGGACAGTGAGG	
RT-IFN-β-F	GCCTTTGCCATCCAAGAGATGC	qPCR for the *IFN-β* gene
RT-IFN-β-R	ACACTGTCTGCTGGTGGAGTTC	
RT-IL-1β-F	TGGACCTTCCAGGATGAGGACA	qPCR for the *IL-1β* gene
RT-IL-1β-R	GTTCATCTCGGAGCCTGTAGTG	

## Data Availability

The original contributions presented in this study are included in the article. Further inquiries can be directed to the corresponding author.

## Supplementary Materials

The following supporting information can be downloaded at: https://www.mdpi.com/article/10.3390/ijms27156585/s1.

## Abbreviations

The following abbreviations are used in this manuscript:

c-di-GMP	Cyclic diguanylate
STING	Stimulator of interferon genes
DGCs	Diguanylate cyclases
PDEs	phosphodiesterases
LPS	Lipopolysaccharide
T4SS	Type IV secretion system
ORF	Open reading frame
TSA	Tryptic soy agar
TSB	Tryptic soy broth
CFU	Colony-forming unit
SDS	Sodium dodecyl sulfate
