# Supplementary material for: Deletion of the c-di-GMP Phosphodiesterase BpdB Attenuates Brucella melitensis Virulence by Enhancing Oxidative Stress Sensitivity, Partially via the Host STING Signaling Pathway
Source: Int J Mol Sci. 2026 Jul 24;27(15):6585. doi: 10.3390/ijms27156585 (PMC13466317; doi:10.3390/ijms27156585)
Supplement: Supplementary file 1 [file ijms-27-06585-s001.zip › ijms-4427100-supplementary.pdf]

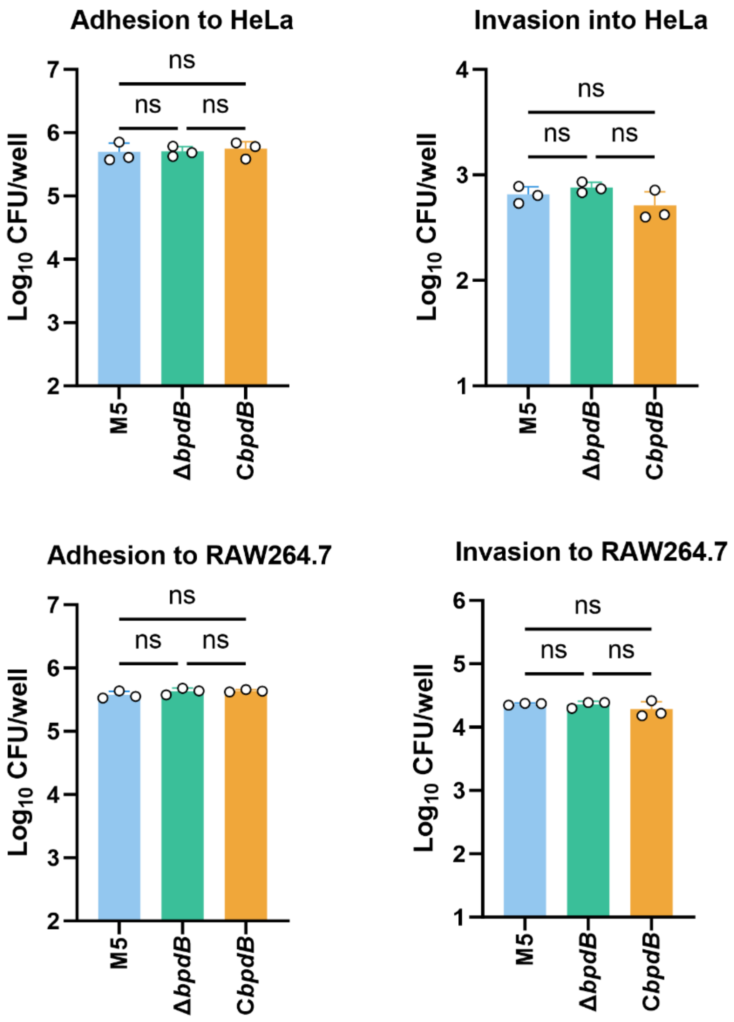

Figure S1. Adhesion and invasion of *Brucella* strains to host cells.
